# Material-Enabled Impact Detection and Damage Localisation System Using Shape Memory Alloy Tufted Composites

**DOI:** 10.3390/s23239565

**Published:** 2023-12-02

**Authors:** Williams Adeyemi, WeeLiam Khor, Francesco Ciampa

**Affiliations:** School of Mechanical Engineering Sciences, University of Surrey, Guildford GU2 7XH, UK; wadeyemi3@gmail.com (W.A.); w.khor@surrey.ac.uk (W.K.)

**Keywords:** structural joints, tufted composited, impact detection, shape memory alloys

## Abstract

Shape memory alloy (SMA) tufted composites have shown a significant improvement of the mechanical strength, fracture toughness, and delamination resistance of structural joints. This paper investigated the self-sensing functionality of SMA tufted carbon/epoxy composite T-joints to enable in situ strain monitoring for the detection of low-velocity impacts. Indeed, large deformations in the tufted composite due to impacts caused abrupt changes in electrical resistance of SMA filaments, which were used to trigger the detection system. An Arduino Mega controller was programmed to simultaneously extract and process real-time electrical resistance recordings from SMA tufts during impact tests conducted at 5 J and 10 J. Experimental results showed that the proposed SMA-enabled detection system can capture accurately the time of the impact and localise the delamination onset, thus demonstrating the truly multifunctional capabilities of proposed SMA tufted composites.

## 1. Introduction

Carbon fibre reinforced polymer (CFRP) composites are widely used in aerospace, automotive and civil engineering applications because of their high strength-to-weight ratio compared to conventional steel and aluminium [1,2,3,4,5]. One of most used geometrical configurations for structural CFRP composite joints is the T-joint design, where the web of the specimen is the structural stiffener, whilst the skin represents the outer shell of the composite part. Nevertheless, despite superior mechanical properties, CFRP joints suffer from damage due to delamination between ply-interfaces, particularly in the web/skin bond line region, leading to low damage resistance. In conventional composite joints, adhesives and mechanical fasteners are used to reduce or prevent the formation of delamination [6,7,8,9,10,11].

Alternatively, some solutions have been explored to mitigate the problem of low out-of-plane delamination resistance, such as the incorporation of foreign materials in the out-of-plane direction as reinforcement material (e.g., stainless-steel [12,13,14,15] and carbon yarns [16,17,18,19,20]). These additions resulted in increased mechanical integrity, where higher delamination resistance was observed [21,22,23,24].

A novel material recently used for the out-of-plane reinforcement of structural composite joints is the shape memory alloy (SMA), whose filaments can be tufted through the thickness of the laminate to improve the mechanical strength, delamination resistance and fracture toughness [20,22,25,26,27,28,29]. SMAs also exhibit the shape memory effect, so that their combined thermo-electrical and thermo-mechanical properties make them suitable for a wide range of applications [30,31,32,33,34,35,36,37]. Studies have shown that by using SMAs for the out-of-plane reinforcement of CFRP composites, in addition to the improvement in delamination resistance, SMAs tufted within the laminate exhibited crack closure properties [21,22,38]. Particularly, the application of electrical current induced the Joule effect in SMA wires, which in turn activated the shape memory effect. Although full closure was not possible due to wedging, the partial closure of the crack and the internal heating could facilitate the repair of the composite [39,40,41]. Despite significant efforts to improve delamination resistance in composites, damage to the material is unavoidable. With this in mind, non-destructive material inspection methods are necessary to enable the engineering critical assessment of composite joining parts.

In real-life operations, continuous and in-service health monitoring of CFRP composites without the need of offline inspection is preferable. Continuous monitoring enables operators to identify damage as it occurs, rather than relying on periodic service checks, which can result in reduced operational downtime and lower costs. In addition to traditional non-destructive methods using external sources, e.g., infrared thermography [42,43,44,45,46] and ultrasonic C-Scan [47,48], sensing devices such as piezoelectric sensors and optical fibres can be integrated into the host composite component to enable continuous damage monitoring [49,50]. One example of material embedded sensing system is the use of optical Fibre Bragg Gratings (FBGs) as strain sensors [51,52,53,54,55,56]. In Takeda et al. [53], small-diameter optical fibres equipped with FBGs were bonded to CFRP composites to ensure that the composite retained the design strength. Signals received by FBG sensors could be displayed on the oscilloscope for visualisation of power levels and wavelengths, which were correlated to the corresponding strain levels. It was found that the maximum amplitude of recorded waves decreased with increasing delamination, which allowed determining the delamination length by measuring the amplitude ratio and the arrival time of new waves. However, the FBG method might not be suitable for impact loads due to the complexity introduced by the combination of failure modes, which could make wave propagation analysis complicated.

An alternative to the use of FBGs for damage detection in CFRP composites subject to structural loading is through the online monitoring of electrical resistance variations of carbon fibres or additional materials integrated into the fibre/matrix lay-up [52,57,58,59,60,61]. An example is the application of electrical current directly in CFRP composites, enabling material-enabled measurements of electrical resistance and capacitance [62,63,64]. In these works, the laminate was subjected to buckling, with copper electrodes attached to fibres at each end of the specimens, thus creating measuring points for the current flow. Resistance measurements from the composite were found to be correlated to strain and damage caused by fatigue [60,62,63,64]. In addition, it was found that the DC current was more sensitive to fibre failures, whilst AC current was more sensitive to matrix failure [63]. Due to variations of electrical resistance across the composite, this technique did require a temperature monitoring system to manage local hot spots on the compote during testing.

By taking advantage of the unique material-enabled capability of CFRP composites to self-sense local strain variations through electrical resistance measurements, this paper explored, for the first time, the potential of employing SMA tufts as detectors of low-velocity impacts in carbon-epoxy joints. SMA were tufted into T-joint composite samples along the web/skin bond line as out-of-plane reinforcements, which successfully improved mechanical performance. These specimens were then instrumented with an Arduino Mega microcontroller to collect resistance measurements from SMA tufts during impact testing using an impact drop tower [65,66,67,68]. Electrical resistance variations measured in SMA tufts were finally recorded and processed to detect low-velocity impacts and to localise the onset of delamination. 

## 2. Methodology

### 2.1. Composite T-Joint Samples

The material used in this study was a biaxial non-crimp fabric (NCF) with a carbon orientation of 0°/90°, along with added toughener veils and a powder binder. Specifications of fibres are described in Table 1.

**Figure 1:**
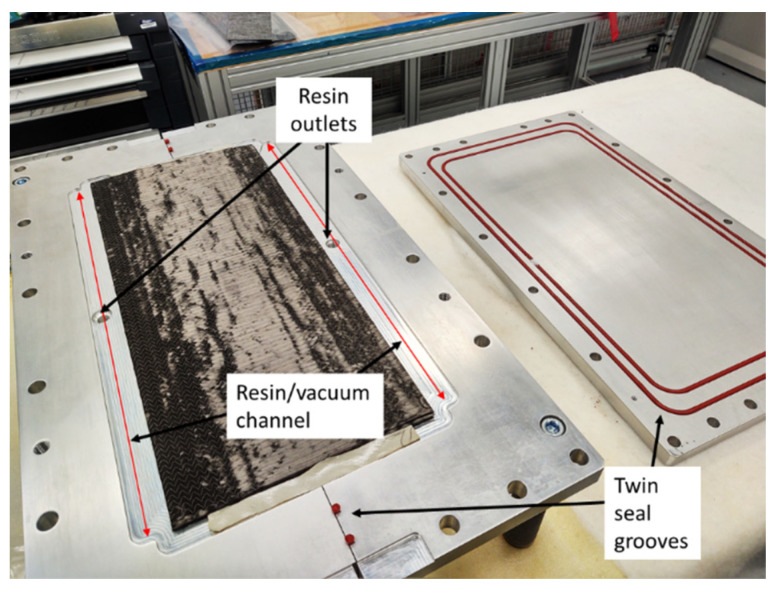
Infusion tooling with preform inserted.

All T-joints were fabricated using the same fibre lay-up, employing three plies for both the web sides and the skin. To fill the void at the centre of the T-joint, a noodle filler composed of a braided UD cord was employed. Preforms underwent a hot debulking process at 120 °C for one hour each, reducing bulk before tufting and facilitating infusion preparation. Carbon preforms were infused with Hexcel RTM 6-2, a two-part aerospace-grade epoxy resin with an operating temperature of up to 120 °C. Each preform was placed within a three-part resin transfer moulding (RTM) infusion tool, comprising a base plate and corner pieces to create the T-shaped geometry, as depicted in Figure 1. The resin underwent a two-hour curing process at 180 °C once the tooling reached this temperature.

**Figure 2:**
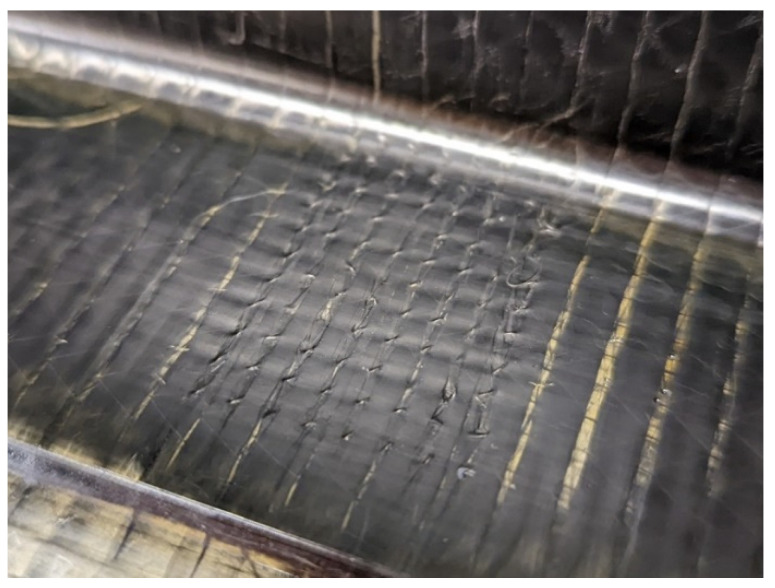
The 3.44 mm spaced tuft loops.

Before infusion, diameter nickel-titanium (Nitinol) SMA tufts with a diameter of 0.13 mm were inserted using a KSL RS 522 tufting head attached to a Kuka KR240 robot, employing a nickel-titanium SMA wire. This procedure was carried out with the preform securely held in specialised tooling, with tufts entering from the skin side and looping on the web side of the T-joint. SMA tufts were inserted in straight, parallel rows perpendicular to the skin surface. The tufted rows were 3.3 mm apart in the length direction and 5.16 mm apart in the width direction, with the loops measuring approximately 2 mm. A study of optimal design for the T-joint with varying tufting parameters was performed by Khor et al. [21]. Tuft loops were kept short to prevent interference between tuft rows. During the tufting process, the formation of the loops generated some plastic deformation within the SMA tufts. Tuft loops were strained to approximately 8.6%, and the bent section of the tuft exhibited a strain of approximately 9%. The austenite finish temperature (*A_f_*) of SMA tufts used in this work is below 0 °C (please note the precise alloy composition and heat treatment of the nitinol is proprietary information). SMAs underwent a phase change from austenite to stressed martensite when deformed above the phase transformation limit (approximately 1% strain). This resulted in a different electrical resistance compared to an unstrained nitinol wire when fitted into the designed system, but it did not affect the analysis. As soon as SMA wires were unloaded, they recovered from the stressed-martensite phase towards the austenite base phase. However, the full recovery was not possible after the impact due to both the pre-stress generated during manufacturing and the fractured composite that inhibited SMAs to return to their original shape. An uncut tufted section with 3.44 mm spacing is displayed in Figure 2.

**Figure 3:**
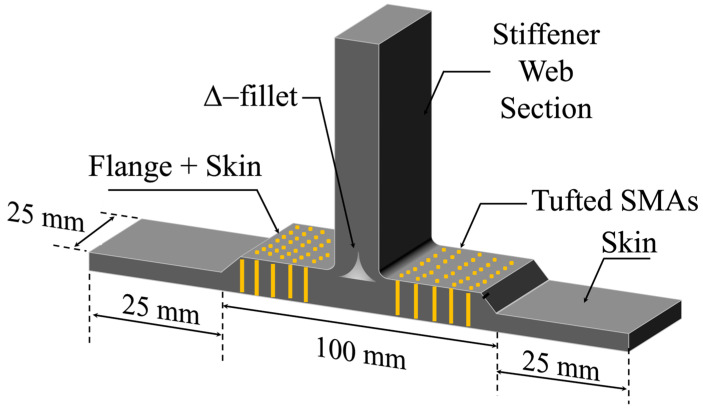
Isometric view of the SMA-tufted composite T-joint.

T-joint sections were extracted using a Sharp & Tappin panel saw. For tufted sections, leads at the start and end of each tuft row were retained and exposed from the resin surface, facilitating strain measurement and resistive heating connections. An illustration of the SMA-tufted T-joint sample is shown in Figure 3.

### 2.2. Multi-Wire Resistance Measurement Using Arduino

SMA wires were used both as reinforcement tufts and embedded sensors within the CFRP T-joint composite. Nitinol’s resistivity undergoes a significant variation as it transitions from the austenite phase, where it measures approximately 100 μΩ cm, to the martensite phase, where it decreases to around 80 μΩ cm. This resulted in distinct electrical conductivity characteristics within these crystallographic phases. It is important to note that the minor difference in resistivity in Nitinol due to these phases did not significantly impact the abrupt change in resistance during impact tests.

**Figure 4:**
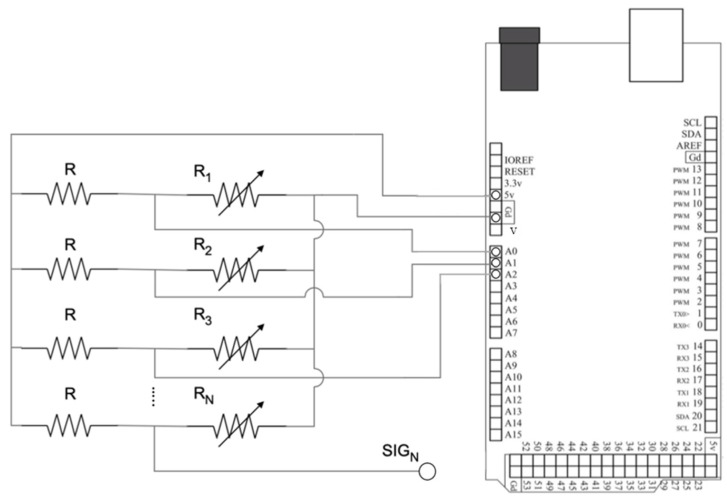
Arduino Mega Rev3 (**right**) connected to the designed system (**left**) with *N* denoting the number of channels on the T-joint structure.

**Figure 5:**
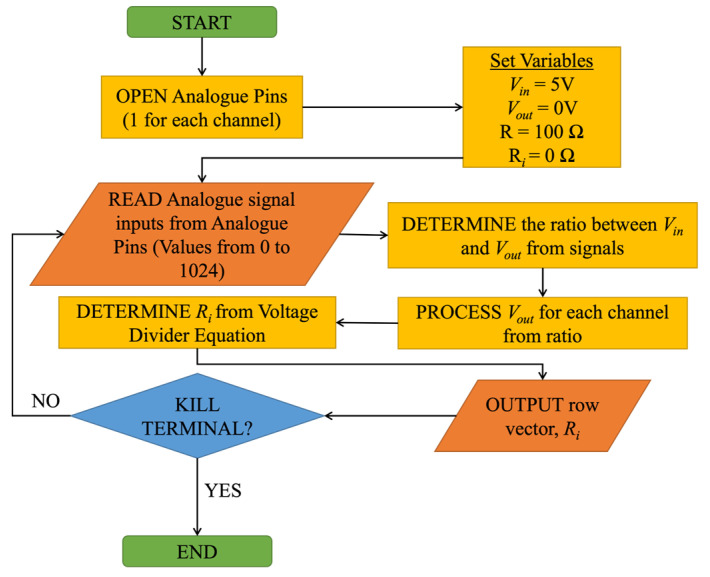
Arduino Flowchart process from receiving the signal to outputting the resultant electrical resistance values, *R_i_*.

The primary objective of the SMA-enable monitoring system was to detect damage in the composite joint caused by the impact event, as well as to retrieve the impact location on the web/skin bond line. The resistance measurement system was constructed using an Arduino Mega Rev3 (Figure 4) and a set of parallel electrical circuits. These electrical circuits consisted of a series of potential divider circuits placed in parallel with each other. Each potential divider circuit was comprised of a 100 Ω fixed resistor (*R*) and variable resistors, $R_{i}\;(i=1,2,\ldots ,N),$ with *N* the number of channels ($C_{1},C_{2},\ldots ,C_{N}$) of the detection system corresponding to the number of SMA tufts. Analogue signals from each potential divider fed to the Arduino Mega with a resolution of 4.88 mV, which was sufficient in this investigation, as the change in voltage was adequate to read small changes in the electrical resistance. The voltage out, *V_out_*, of the circuit was given as a decimal ranging from 0 to 1024, corresponding to 0 V–5 V. Based on this relationship, nitinol wires acted as strain sensors upon deformation and the resistance of each SMA wire could be deduced using Equation (1) below. Variations of nitinol wires’ resistance values, *R_i_*, are given by,

*V_out_* = [*R*/(*R* + *R_i_*)]*V_in_*,

*R_i_* = [(*V_in_*/*V_out_*) − 1] × *R*, *i* = 1, 2,..., *N*, (1)

where *V_in_* is the input voltage of the circuit. Multicore 24 AWG wires were soldered to the SMA wires to establish an electrical connection between tufts and the circuit. Equation (1) was programmed into the Arduino UI to deduce the resistance measurement from each SMA wire. Figure 5 illustrates the flowchart for the extraction process of electric resistance values, *R_i_*, from each nitinol wire.

A total of 10 channels were set up to simultaneously collect measurements from 10 SMA wires. The maximum measurement of the system was set to 200 Ω for practicality purposes. Considering that the Arduino system runs at *V_in_* = 5 V, the power consumed by each channel was predicted to be at 83.3 mW. Details of the operating condition of the Arduino system are described in Table 2.

### 2.3. Impact Testing of T-Joints

**Figure 6:**
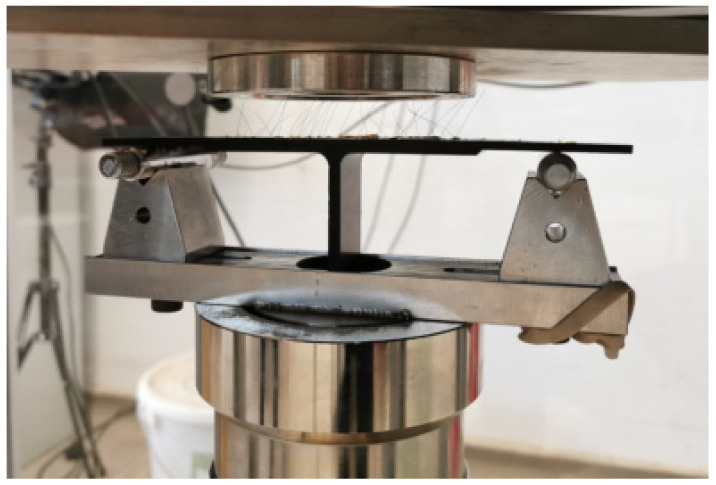
Simply supported SMA tufted T-joint specimen in the impact machine during drop-weight testing.

Composite T-joints were tested using an Instron 9450 drop-weight impact machine in a simply supported configuration, as depicted in Figure 6. When the impactor struck the centre of the specimen, mechanical stresses resulted in the formation of material damage and delamination, leading to the straining of SMA tufts and the corresponding abrupt changes of their electrical resistance. Two levels of impact energy were subsequently applied to each test specimen, i.e., 5 J and 10 J at velocities of 1.37 m/s and 1.97 m/s, respectively. The impact was directed at the centre of the web/skin bond line region, above the stiffener. A total of *N* = 10 channels, C*_i_* (i=1,2,…,N), were monitored concurrently, corresponding to 10 SMA wires across T-joint specimens. Each wire and connection were insulated using cello tape to ensure that there was no interference due to short circuits. A total of three T-joint specimens were tested.

## 3. Results and Discussion

**Figure 7:**
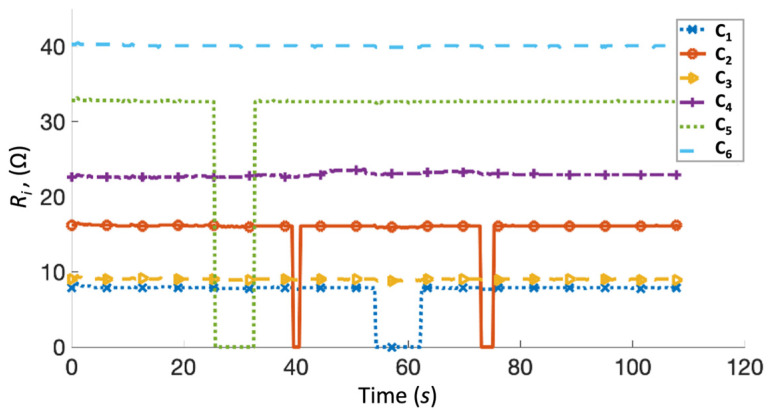
Multiple resistance channels being detected simultaneously where some channels were disconnected and reconnected to simulate broken SMA tufts.

An initial test was performed on the Arduino resistance measurement system using fixed resistors for the performance validation. Fixed resistors represented the tufted SMA wire embedded within the composite, and a switch was connected in series to simulate disconnection due to the total breakage of the SMA wire. A total of six channels were activated on the Arduino system, with a resistor ranging from 8 to 40 Ω connected to each channel. The maximum measurement of the system was set to 200 Ω, significantly higher than the initial resistance of the SMA wire in an undamaged composite, which is approximately 10 Ω. Resistance measurements from each channel of the Arduino system were recorded for 110 s, as shown in Figure 7.

**Figure 8:**
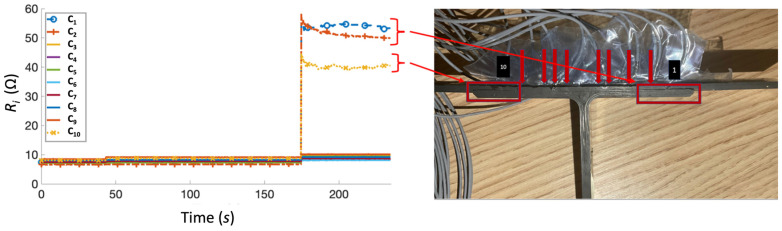
Test 1 of the test specimen (**right**) after impact test and the corresponding resistance versus time graph (**left**). Arrows indicate location of SMA tuft across specimen width.

The resistance measured during the initial test was consistent with the connected fixed resistors, with an error of ±8%. This is considered acceptable since the main purpose of the system was to detect changes in electrical measurements, rather than function as a pure multimeter. The switch was triggered to break circuits on channels C_1_, C_2_, and C_5_ during the test, and the system responded with measurements of 0 Ω resistance for the open circuits. Three composite T-joint specimens were then tested using the impact machine. In the illustration of composite samples, locations of SMA wires were labelled 1 and 10, with red arrow indicating the location of wires 2 to 9 (Figure 8). In each test, a 5 J impact was first applied, followed by a 10 J impact.

For the first specimen, the initial 5 J impact was executed at the time *t* = 48 s, followed by 10 J at *t* = 174 s. During the first impact, minimal increases in resistance were measured on channels C_9_ and C_10_, corresponding to the left outermost wires (Figure 8), suggesting that the first delamination initiated from the left side of the specimen. During the second impact, a significant increase in resistance was detected on channels C_1_ (right outermost), C_2_, and C_10_ (left outermost). This detection is consistent with the onset of delamination observed on both sides of the specimen during impact testing (Figure 8). All other channels showed an ~2 Ω increase in resistance measurements, suggesting that all wires in the specimen underwent minimal deformation straining.

**Figure 9:**
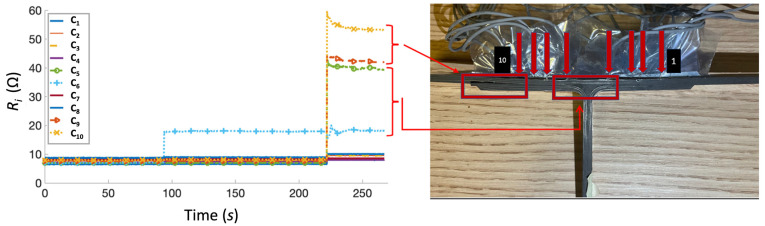
Test 2 of the test specimen (**right**) after impact test and the corresponding resistance versus time graph (**left**). Arrows indicate location of SMA tuft across specimen width.

The second specimen was first impacted at the time *t* = 94 s, followed by a second impact at *t* = 222 s (Figure 9). During the first impact, damage was detected by the channel C_6_, around the middle of the specimen. At the second impact, channels C_10_, C_9_, and C_5_ showed a significant spike in resistance measurements, while a tiny fluctuation was observed in C_6_. This suggests that initial delamination occurred in the middle of the specimen, around the noodle and web area. Similar to Figure 8, electrical resistance variations measured in these channels were consistent with damage observations on the specimen, since some delamination occurred in the middle of the sample, whereas major delamination originated on the left side of the T-joint. As before, all other channels showed only an approximately 2 Ω increase in resistance.

**Figure 10:**
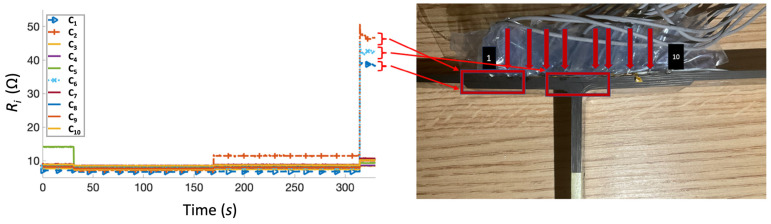
Test 3 of the test specimen (**right**) after impact test and the corresponding resistance versus time graph (**left**). Arrows indicate location of SMA tuft across specimen width.

On the third specimen, the first 5 J impact was applied at the time *t* = 170 s, followed by a 10 J impact at *t* = 314 s (Figure 10). During the first impact, the channel C_2_ showed a significant increase in the measured resistance, suggesting that delamination initiated from one end of the specimen. During the second impact, channels C_1_ (leftmost), C_2_, and C_6_ (middle) exhibited a significant spike in resistance measurements, consistent with the delamination observed on the left and middle of the specimen. As above, approximately a 2 Ω increase was observed in all channels after the second impact.

In all specimens, the spikes in resistance measurements were consistent with the onset of delamination observed in T-joints during impact testing. Prior to testing, the specimens were nominally undamaged and did not have any artificial weak spots designed into them. In the first test, delamination was observed on both sides of the specimen, while in the second and third tests, delamination was seen at one end and in the middle of the specimens. The location of the onset of delamination was likely determined by local weak spots within the composite. It is advised, instead, that composite specimens intentionally designed with weak spots for damage initiation, such as the incorporation of Teflon patches within the composite, would result in a consistent damage initiation area during impact tests. In all T-joint specimens, there was a consistent increase of approximately 2 Ω across all channels, particularly after the second impact at 10 J. This suggests that the bending of the specimen due to the impact caused permanent straining of SMA tufts in the out-of-plane direction, even though no damage was observed on the surface of the composite after the tests. This shows that tufted SMAs can indicate strain or damage experienced by the composite and the onset of delamination, despite the appearance on the surface of the material.

Therefore, the introduction of SMA tufting to join CFRP composite parts would facilitate the development of a lightweight and damage tolerant joint, as well as provide local capability for online damage assessment post-manufacture and throughout service life. In the aviation sector, for example, mechanical fasteners are available in large quantities in the composite wing ribs of the aircraft 787-Dreamliner [69,70,71,72,73]. The proposed technology has the potential to replace fasteners and significantly reduce aircraft maintenance downtime and inspection costs.

## 4. Conclusions

The electrical conductivity of out-of-plane SMA tuft reinforcement in composites showed potential for damage detection and localisation. An Arduino-based resistance measurement system was developed to simultaneously measure ten electrical parameters from a composite T-joint specimen during impact tests conducted at 5 J, followed by 10 J of impact energy. Spikes in resistance measurements from the Arduino system were consistent with the locations where delamination was observed in the T-joint specimens. Additionally, an approximately 2 Ω increase in resistance was observed across all three specimens after the impact with 10 J impact energy, suggesting that the SMA wires were sensitive to measurement of minor straining in the composite despite no visible damage observed on the sample surface.

## Figures and Tables

**Table 1:** Fibre construction of NCF.

Layer	NCF Material	Areal Weight (g/m^2^)
Powder Binder	Solvay Cycom 7720	10
Toughening Veil	TA1903	4
Fibre	Tenax-E ITS55 E23 24K 1600tex, 90°	268
Toughening Veil	TA1903	4
Fibre	Tenax-E ITS55 E23 24K 1600tex, 0°	268

**Table 2:** Operating conditions of the Arduino system.

Parameter	Value
Number of channels, *N*	10
Total input voltage, *V_in_*	5 V
Maximum resistance measurement on *R_i_*	200 Ω
Fixed resistance, *R*	100 Ω
Maximum power per channel at *R*_2_	83.3 mW
Maximum total power of system	833 mW

## Data Availability

The script for the Arduino system can be provided upon request.
